# Electroacupuncture for treating cancer-related insomnia: a multicenter, assessor-blinded, randomized controlled, pilot clinical trial

**DOI:** 10.1186/s12906-022-03561-w

**Published:** 2022-03-18

**Authors:** Boram Lee, Bo-Kyung Kim, Mikyung Kim, Ae-Ran Kim, Hyo-Ju Park, O-Jin Kwon, Jun-Hwan Lee, Joo-Hee Kim

**Affiliations:** 1 Clinical Medicine Division, Korea Institute of Oriental Medicine, Daejeon, Republic of Korea; 2 Department of Neuropsychiatry, School of Korean Medicine, Pusan National University, Yangsan, Republic of Korea; 3 Department of Internal Medicine, College of Korean Medicine, Sangji University, Wonju, Republic of Korea; 4 Korean Medicine Life Science, University of Science & Technology (UST), Campus of Korea Institute of Oriental Medicine, Daejeon, Republic of Korea; 5 Department of Acupuncture and Moxibustion Medicine, College of Korean Medicine, Sangji University, Wonju, Republic of Korea; 6 Research institute of Korean medicine, Sangji University, Wonju, Republic of Korea

**Keywords:** Electroacupuncture, Cancer-related insomnia, Sleep initiation and maintenance disorders, Randomized controlled trial

## Abstract

**Background:**

Insomnia is one of the most frequent symptoms in people with cancer. Electroacupuncture has been widely used in people with cancer or insomnia. We explored the feasibility and preliminary effectiveness of electroacupuncture for cancer-related insomnia.

**Methods:**

People with cancer and insomnia disorder were randomly allocated to electroacupuncture, sham-electroacupuncture, or usual care groups. Participants received either 10 sessions of electroacupuncture at real acupoints, sham-electroacupuncture at non-acupoints, or usual care in each group for 4 weeks. We calculated the recruitment, adherence, and completion rates of participants. The Insomnia Severity Index (ISI), Pittsburgh Sleep Quality Index (PSQI), sleep diary and actigraphy-derived sleep parameters, Functional Assessment of Cancer Therapy-Fatigue (FACT-F), Montreal Cognitive Assessment (MoCA), and salivary levels of cortisol and melatonin were evaluated as outcome measures.

**Results:**

Twenty-two participants were enrolled (8, 6, and 8 respectively in the electroacupuncture, sham-electroacupuncture, and usual care groups) and 20 participants completed the trials (8, 4, and 8 respectively). The recruitment, adherence, and completion rates were 78.57% (22/28), 95.45% (21/22), and 90.91% (20/22), respectively. Most of the participants had previously received conventional treatment for insomnia, but few had received Korean medicine treatment, without any demographic or clinical differences between groups. In the electroacupuncture group, there was a statistically significant reduction of 10.13 (mean) ± 8.15 (standard deviation) and 5 ± 3.70 points in mean ISI and PSQI scores at 4 weeks post-treatment (*P* = .0098 and .0066), compared with sham-electroacupuncture (2.06 ± 7.15 and 1.61 ± 4.34; *P* = .4796 and .3632) and usual care (3.25 ± 2.60 and 1.38 ± 2.13; *P* = .0096 and .1112). Although there was no significant difference in ISI score between groups at 4 weeks post-treatment, the electroacupuncture group continued to improve significantly at 4 weeks’ follow-up, showing borderline and significant differences compared to the sham-electroacupuncture and usual care (*P* = .0614 and .0015). The FACT-F scores in electroacupuncture group showed a significant improvement compared with the sham-electroacupuncture group (*P* = .0305). No electroacupuncture-related adverse events were reported.

**Conclusions:**

Electroacupuncture might be feasible for cancer-related insomnia, despite slow participant recruitment. Additional trials with adequately powered sample sizes and a substantial change to the recruitment procedure are needed.

**Trial registration:**

Clinical Research Information Service, KCT0002162. Submitted 27 October 2016, Registered 2 December 2016 - Retrospectively registered (The first participant enrolment: 28 November 2016),

**Supplementary Information:**

The online version contains supplementary material available at 10.1186/s12906-022-03561-w.

## Background

The prevalence of insomnia in people with cancer is estimated to be over 60% [1, 2], 2–3 times more than that in the general population [3]. This high incidence can be attributed to the emotional consequences of a cancer diagnosis as well as the adverse effects of cancer treatments such as chemotherapy [3, 4]. Although medical and psychological parameters such as distress and depression tend to improve after cancer treatment, insomnia in people with cancer tends to be highly persistent [5]. Insomnia is known to negatively influence the quality of life and pain in people with cancer [6]. It can also affect the natural history of a tumor by suppressing the immune response [7]. Therefore, the National Cancer Institute (NCI) encourages cancer survivors to discuss chronic sleep disturbances with their medical team during the course of routine survivorship care [8].

The standard therapy for insomnia is Cognitive Behavior Therapy for Insomnia (CBT-I) and pharmacotherapy. Although CBT-I is strongly recommended in people with cancer [9], the low adherence and the lack of response in a significant proportion of patients are important issues [10, 11]. In addition, there are limited data on the efficacy of hypnotics in people with cancer, and polypharmacy is preferably reduced as much as possible in cancer treatment to reduce potential drug-drug interactions [12].

Acupuncture and electroacupuncture (EA), a combination of acupuncture and electric stimulation [13], are widely used to the improve quality of life of people with cancer and provide support during conventional treatment such as chemoradiotherapy [14]. About a third of people with cancer in European countries and 1 in 10 people with cancer in the United States have used acupuncture, with the acupuncture use rate among people with cancer being higher than that in people without cancer [15, 16]. Furthermore, about 59% of NCI-designated cancer centers incorporate acupuncture for the management of cancer [17].

A recent systematic review concluded that the level of evidence of acupuncture for cancer-related insomnia is low because of the small number of studies included and their low methodological quality [18]. However, among the randomized controlled trials (RCTs) included in this review, there were only two previous studies [19, 20] that recruited participants with both cancer and insomnia, and most included studies have evaluated insomnia symptoms in people with cancer regardless of whether they had insomnia symptoms to assess their quality of life or neuropsychiatric status. In addition, RCTs targeting cancer-related insomnia compared acupuncture with conventional drugs [19, 20], and they used the efficacy rate calculated based on sleep efficiency, a non-validated secondarily-processed outcome, as the primary outcome. Moreover, in both studies, the treatment duration was as short as 7 days, so only the short-term effect of acupuncture treatment was measured. Thus, to the best of our knowledge, there is no RCT targeting the clinical efficacy and safety of EA in comparison with sham-EA using validated sleep-related outcome measures for cancer-related insomnia. This study aimed to explore the feasibility and preliminary effectiveness of EA in people with cancer-related insomnia using both subjective and objective sleep measures.

## Methods

### Study design

We conducted a 3-arm, multicenter, randomized, assessor-blinded trial comparing EA, sham-EA, and usual care from October 2016 to January 2018 at Daejeon Korean Medicine Hospital of Daejeon University and Dongeui University Korean Medical Hospital in Republic of Korea. This study was approved by the institutional review board at each Korean medicine hospital (approval numbers: DJDSKH-17-BM-06 and 2016–04) and registered at the Clinical Research Information Service (KCT0002162). The detailed study protocol has been previously published [21]. Due to a lack of previous studies, we could not find adequate references for a sample size calculation and therefore this pilot study was undertaken to provide a reference for this calculation. Considering the recommendation that the minimal number of participants in each group for a pilot study is 12 [22], a sample size of 15 participants (considering a 20% drop-out rate) in each group was determined to be adequate for assessing feasibility.

### Study participants

Participants were recruited through advertisements such as clinical trial institutions websites and local newspapers. The inclusion criteria were (1) age, 19–79 years; (2) Eastern Cooperative Oncology Group performance status ≤2, (3) cessation of cancer-related treatments at least 12 weeks prior to enrolment (ongoing hormone therapy was allowed if it was initiated from at least 3 weeks before the trial), (4) cancer-related insomnia for at least 3 months, meeting the criteria for insomnia disorder of the Diagnostic and Statistical Manual of Mental Disorders, Fifth Edition, (5) Insomnia Severity Index (ISI) score ≥ 15, and (6) voluntary provision of written informed consent.

We excluded persons who (1) had experienced the current level of insomnia prior to cancer diagnosis, (2) had severe anemia, (3) were diagnosed as having psychiatric disorders; caffeine, alcohol, or drug addiction; or scores ≥11 on the anxiety or depression subscale in hospital anxiety and depression scale, (4) had cancer-related pain rated ≥4 on the numeric rating scale, (5) had an estimated life expectancy ≤6 months, (6) were scheduled to undergo radiotherapy, or chemotherapy during the trial period, (7) had changed their regular conventional medication to alleviate insomnia within 4 weeks, (8) had taken sedative hypnotics as needed to improve insomnia within 2 weeks, (9) had received Korean medicine treatments such as acupuncture within 4 weeks, (10) had initiated or changed dietary supplement regimens or non-pharmacological therapeutic regimens such as CBT-I within 4 weeks, (11) were working shifts or had variable day/night work schedules that could affect circadian rhythm, (12) were suffering from pain or any disease severe enough to cause sleep disturbances, (13) were taking blood coagulants, (14) had abnormal levels of free thyroxine [free T4] and thyroid-stimulating hormone [TSH] < 0.1 μIU/mL or > 5.1 μIU/mL, (15) had experienced hypersensitivity reactions to acupuncture or inability to cooperate with acupuncture treatment, (16) had implants that could interfere with EA, (17) were pregnant, lactating, or planning to become pregnant, (18) had participated in another clinical trial within 4 weeks, or (19) had difficulty in complying with the trial protocol [21].

### Randomization and blinding procedures

Using SAS version 9.4 (SAS Institute Inc., Cary, NC, USA), a statistician who was blinded to the intervention and evaluation generated random sequence numbers, with an allocation ratio of 1:1:1 to the 3 groups. A stratified block randomization method was used, with the research center and the provision of standard therapies for insomnia, such as CBT-I or hypnotics, as stratification factors. For allocation concealment, we used sealed opaque envelopes and kept them in double-locked cabinets at each center. Each envelope was opened in sequence by the investigators at the baseline visit of the enrolled participants. The participants in the usual care group and acupuncture practitioners could not be blinded due to the nature of the intervention. The outcome assessors and data analysts were blinded because they were not involved in the treatment procedures.

### Intervention

Using a brochure, all participants received sleep hygiene education. The EA group received acupuncture treatment with disposable sterilized acupuncture needles (0.25 × 25 mm; Asiamed, Germany) at GV20, EX-HN3, bilateral HT7, PC6, BL63, and KI4 (depth of needle insertion: 0.2–1.5 cun). In addition to these 10 acupoints, up to 4 acupoints were optionally added depending on the individual symptoms of the participant. After achieving the “de qi” sensation by twisting the needles, an EA device (ES-160; Ito Co. Ltd., Tokyo, Japan) was used for electrostimulation with a 4-Hz frequency and continuous wave for 30 min/time, 2–3 times a week for 4 weeks, for a total of 10 sessions. The treatment regimen (acupoint, acupuncture stimulation method, and frequency and duration of treatment, etc.) was based on previous EA studies on insomnia and the consensus among Korean medicine specialists [23, 24].

In the sham-EA group, a placebo Streitberger acupuncture needle (Asiamed, Germany), with a blunt end not penetrating the skin [25, 26], was inserted at 10 non-acupoints, the same number as in the EA group, unrelated to the management and treatment of sleep disorders in Korean medicine theory on both the arms and legs: “Arm 1” (1 cm lateral and 5 cm proximal points from the cubital creases of both arms), “Arm 2” (2 cm above “Arm 1”), “Leg 1” (1.5 cm above the middle of the patella), “Leg 2” (upper 1/3rd of the medial part of the tibia), and “Leg 3” (1.5 cm below “Leg 2”). A de-activated EA device was connected to the Streitberger acupuncture needle, making the same beeping sound and light indicators without delivering any electrostimulation. Other treatment regimens were identical to those in the EA group. Treatment procedures in the EA and sham-EA groups were conducted by Korean medicine doctors with at least 2 years of clinical experience and at least 6 years of the regular curriculum of medical college. In addition, to ensure the quality of the clinical trial, a workshop for standardized acupuncture treatment was conducted so that there was no difference in acupuncture treatment between acupuncture practitioners and clinical trial institutions before the trial initiation.

In the usual care group, participants received the usual treatment and performed self-care. For all participants in the three groups, if the participants had regularly received conventional insomnia treatments such as hypnotics or CBT-I for at least 4 weeks prior to study initiation, they were allowed to continue doing so during the clinical trial unless their treatment regimen was changed. No additional treatments for insomnia other than those permitted at the screening visit were allowed during the trial period.

### Outcome measures

The recruitment rate (number of enrolled participants/number of screened participants), completion rate (number of participants who completed the clinical trial/number of enrolled participants), and adherence rate (number of participants who completed at least 7 of 10 treatment sessions/number of enrolled participants), were calculated to check the feasibility of a confirmatory clinical trial. The primary outcome was the ISI score at 4 weeks post-treatment. The ISI is a validated, self-administered questionnaire assessing the insomnia severity [27]. It is also a reliable and valid measure of insomnia in people with cancer [28]. The total ISI score ranged from 0 to 28, with higher scores indicating severe insomnia. The validated Korean version of ISI was used in this study [29].

The secondary outcome measures included (1) the ISI score at 2 weeks post-treatment and 4 weeks of follow-up, (2) Pittsburgh Sleep Quality Index (PSQI) score, (3) sleep quality measured by sleep diary and actigraphy, (4) Functional Assessment of Cancer Therapy-Fatigue (FACT-F) score, (5) Montreal Cognitive Assessment (MoCA) score, and (6) the levels of salivary cortisol and melatonin.

The PSQI is a self-rated questionnaire that assesses sleep quality and disturbances within the past month by measuring subjective sleep quality, sleep-onset latency (SOL), total sleep time (TST), sleep efficiency (SE), sleep disturbances, use of sleeping medication, and daytime dysfunction [30, 31]. A total score of more than 5 out of 21 indicates a significant sleep disturbance [30]. Sleep diary assesses bedtime and rising time, from which the total time in bed (TIB) was calculated. It also assesses SOL and TST. We calculated the sleep diary-derived SE as (TST/TIB × 100%). We compared the mean change in the sleep diary parameters during 1 week before each visit. The actigraph (ActiGraph WGT3X-BT; MT1 Health Services Company, Pensacola, Florida, USA) was worn on the non-dominant wrist during the week before each visit to objectively measure sleep patterns. We analyzed the objective sleep quality data, including SOL, TST, and SE, from actigraphy at 2 and 4 weeks post-treatment and 4 weeks of follow-up. In addition, the correlation of SE values extracted from actigraphy and the sleep diary was analyzed using Pearson correlation coefficient.

The FACT-F is a self-report questionnaire designed to measure the level of fatigue in people with cancer and is the most commonly used scale for measuring cancer-related fatigue [32, 33]. It consists of 27 items in FACT-general and an additional 13 items of the fatigue subscale scored from 0 to 160, with higher scores suggestive of less fatigue. The MoCA is a 30-point cognitive screening test originally designed to identify mild cognitive impairment, with higher scores indicating better cognition [34]. Several studies have also explored its applicability to insomnia in people with cancer [35]. The levels of melatonin usually surge at night, while cortisol levels increase in the morning in normal conditions [24, 36]. According to previous studies, acupuncture can improve insomnia by influencing the secretion of cortisol [37, 38] and melatonin [39]. We evaluated the levels of salivary cortisol and melatonin by EA before and 4 weeks after treatment to observe whether these rhythmical changes showed any differences in people with cancer-related insomnia. A saliva tube with an absorbent swab (Green Cross LabCell, Yongin, Republic of Korea) was distributed to the participants in advance, and they were instructed to self-collect saliva samples before bedtime (10 PM) the day before visit days (baseline and 4 weeks post-treatment) and after waking up (7 AM) on these visit days. To ensure the sufficient absorption of saliva, the participants were instructed to use their molar teeth to bite the absorbent swab tightly. Then, the participants put the swab into the tube and immediately stored it in a frozen state stably. To maintain the stability of the specimen, the investigators visited each study participant’s house on the scheduled visit days and transported the samples in an opaque silver foil cold pack to the clinical institution in a frozen state using an icebox. The samples were stably stored in a frozen state (below − 60 °C) in each clinical institution, transported to a designated central laboratory (Green Cross LabCell, Yongin, Republic of Korea), and analyzed collectively using enzyme-linked immunosorbent assay methods. To perform accurate tests and obtain stable samples, the participants were educated to restrict alcohol, caffeine, and nicotine intake up to 12 h before sample collection and not to eat, drink, or brush their teeth until 1 h before sample collection. In addition, the researcher distributed educational brochures detailing the sample collection method, collection time, and precautions to the participants and conducted sufficient training in collection methods.

For the safety assessment, the researchers used data from medical examinations and laboratory tests to investigate all adverse events (AEs) during the trial period, irrespective of their relationship with the intervention. The AEs including nervous system disorders, gastrointestinal disorders, and cardiac and vascular disorders can occur after EA [40]. If the Korean medicine doctor judged such AEs to have a causal relationship with the EA treatment by the Korean medicine doctor in terms of the onset and disappearance time of symptoms, participant’s usual condition, and past history, it was judged as an AE related to the EA.

After the first and last treatments, blinding and credibility tests were performed in the EA and sham-EA groups. To check whether participant blinding was maintained, the participants were asked to guess the type of treatment they received: “real EA,” “sham-EA,” or “unknown.” The success of blinding was assessed using the new blinding index (BI) and 95% confidence interval (CI) [41]. The credibility test assessed the level of confidence in the treatment the participants received using 4 items. The score for each item ranged from 0 to 6, with higher scores reflecting higher confidence.

### Statistical analysis

A statistician conducted a statistical analysis based on a full analysis set using SAS version 9.4 software (SAS Institute Inc., Cary, NC, USA). A two-sided test with a significance level of 0.05 was conducted by an analysis of covariance (ANCOVA) with baseline as the covariate. A paired t-test or Wilcoxon signed rank test was used to analyze the changes in the results before and after treatment within the groups. The multiple comparison problem was solved using the fixed sequence method and a repeated-measures analysis of variance with a post-hoc Dunnett’s procedure was used to validate the between-group differences over time. The multiple imputation method was adopted for missing data.

## Results

### Study participants

**Fig. 1:**
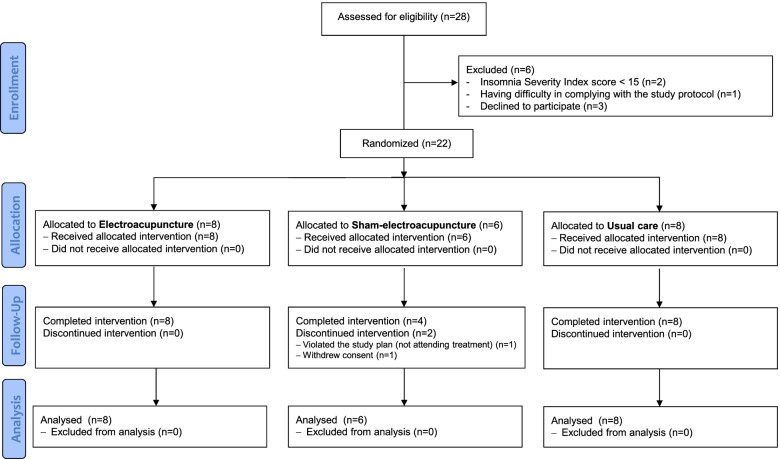
Study flowchart

A total of 28 participants were screened for eligibility during the trial period, of which 22 were randomly assigned to the EA (*N* = 8), sham-EA (*N* = 6), and usual care (*N* 8) groups (recruitment rate, 78.57%). Of these, 20 completed the study and 2 dropped out, all from the sham-EA group. One participant withdrew the consent to participate in the trial because of personal reasons not related to the acupuncture procedure and research and the other violated the study protocol by not attending treatments (Fig. 1). In Daejeon Korean Medicine Hospital of Daejeon University, three participants were screened, of which two were enrolled and completed the trial. In Dongeui University Korean Medical Hospital, twenty-five participants were screened, of which 20 were enrolled and 18 completed the trial. The adherence rates in the EA, sham-EA, and usual care groups were 100% (8/8), 83.33% (5/6), and 100% (8/8) (overall, 95.45% [21/22]), and the completion rates were 100% (8/8), 66.67% (4/6), and 100% (8/8) (overall, 90.91% [20/22]). In addition, all enrolled participants collected saliva samples well during the trial period. The baseline demographic and clinical characteristics are shown in Table 1. No statistically significant baseline between-group differences were observed (all, *P* > .05). Most of the participants were between 50 and 70 years old, and most did not smoke or drink alcohol. The participants’ cancer type varied, including breast, thyroid, and gastrointestinal cancers. Most of the participants had previously received conventional treatment for insomnia, but few had received Korean medicine treatment before participating in the clinical trial.

**Table 1:** Demographic and Clinical Characteristics

Characteristics^**a**^	Electroacupuncture (***N*** = 8)	Sham (***N*** = 6)	Usual-care (***N*** = 8)	***P*** value
Gender (Male/Female)^b^	2 (25.0%) / 6 (75.0%)	5 (83.3%) / 1 (16.7%)	3 (37.5%) / 5 (62.5%)	0.1286
Age (year)^c^	57.63 (52.53, 62.72)	62.33 (54.43, 70.23)	61.38 (55.12, 67.63)	0.4128
Height (cm)^c^	161.02 (155.40, 166.65)	167.33 (162.95, 171.72)	161.38 (152.90, 169.85)	0.2729
Weight (kg)^c^	59.78 (49.99, 69.56)	62.83 (56.42, 69.25)	61.50 (49.33, 73.67)	0.8884
BMI (kg/m^2^)^c^	22.93 (19.86, 25.99)	22.39 (21.09, 23.69)	23.42 (20.15, 26.70)	0.8476
Smoke (Yes/No)^b^	0 (0.0%) / 8 (100.0%)	1 (16.7%) / 5 (83.3%)	0 (0.0%) / 8 (100.0%)	0.2727
Drink (Yes/No)^b^	0 (0.0%) / 8 (100.0%)	2 (33.3%) / 4 (67.7%)	0 (0.0%) / 8 (100.0%)	0.0649
Duration of symptom (Month)^c^	29.54 (0.00, 63.74)	24.90 (8.54, 41.26)	56.21 (15.77, 96.65)	0.2758
Treatment expectation^c,d^	6.13 (4.91, 7.34)	6.83 (5.80, 7.87)	6.75 (4.44, 9.06)	0.7504
Primary tumor^b^				0.2552
Breast	3 (37.5%)	0 (0.0%)	3 (37.5%)	
Thyroid	2 (25.0%)	1 (16.7%)	1 (12.5%)	
Gastrointestinal	0 (0.0%)	2 (33.3%)	3 (37.5%)	
Others	3 (37.5%)	3 (50.0%)	1 (12.5%)	
TNM stage^b^				0.2654
I	3 (37.5%)	2 (33.3%)	4 (50.0%)	
II	1 (12.5%)	2 (33.3%)	1 (12.5%)	
III	0 (0.0%)	0 (0.0%)	2 (25.0%)	
IV	0 (0.0%)	1 (16.7%)	1 (12.5%)	
Unknown	4 (50.0%)	1 (16.7%)	0 (0.0%)	
Insomnia treatment history^b^				
Conventional treatment (Yes/No)	5 (62.5%) / 3 (37.5%)	5 (83.3%) / 1 (16.7%)	5 (62.5%) / 3 (37.5%)	0.7242
Korean medicine treatment (Yes/No)	0 (0.0%) / 8 (100.0%)	0 (0.0%) / 6 (100.0%)	1 (12.5%) / 7 (87.5%)	0.9999
ECOG-PS^b^				0.8529
0	2 (25.0%)	3 (50.0%)	3 (37.5%)	
1	6 (75.0%)	3 (50.0%)	5 (62.5%)	

*Abbreviations*: *BMI* body mass index, *ECOG-PS* Eastern cooperative oncology group-performance status, *TNM* tumor, node and metastasis classification

^a^Data are presented as mean and 95% confidence interval or number (%)

^b^Fisher’s exact test

^c^Analysis of variance test

^d^Self-report questionnaires are used on a scale of 1 to 9, with higher scores indicating higher expectations

### Primary and secondary outcomes

**Fig. 2:**
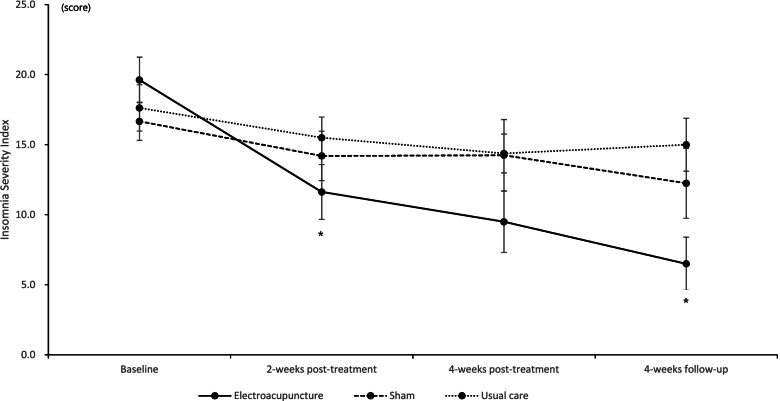
Mean Insomnia Severity Index scores of the 3 groups. *significant difference between electroacupuncture and usual care group, *P* < .05

In the mean ISI score, there was significant reduction of 8 and 10.13 points at 2 and 4 weeks post-treatment in the EA group (*P* = .0093 and *P* = .0098), with lower reduction in sham-EA (2.25 and 2.06 points) and usual care groups (2.13 and 3.25 points). The difference between EA and sham-EA was not significant (*P* = .2219). There was borderline significance (*P* = .0639) between EA and usual care at 4 weeks post-treatment. However, only the EA group continued to improve significantly at 4 weeks’ follow-up (*P* = .0004), showing borderline and significant differences compared to the sham-EA (*P* = .0614) and usual care (*P* = .0015) (Fig. 2, Table 2).

**Table 2:** Subjective Sleep Measures

Variable	Electroacupuncture (*N* = 8)		Sham (*N* = 6)		Usual-care (*N* = 8)		*P* value (Electroacupuncture versus sham)	*P* value (Electroacupuncture versus usual-care)
	Mean (95% CI)	*P* value (within group)	Mean (95% CI)	*P* value (within group)	Mean (95% CI)	*P* value (within group)		
ISI								
Baseline	19.63 (15.78, 23.47)		16.67 (13.18, 20.16)		17.63 (13.73, 21.52)			
2-weeks post-treatment	11.63 (7.00, 16.25)	**0.0093**	14.41 (10.75, 18.07)	0.2581	15.50 (12.01, 18.99)	**0.0310**	0.2422	**0.0481**
4-weeks post-treatment	9.50 (4.33, 14.67)	**0.0098**	14.60 (9.69, 19.51)	0.4796	14.38 (11.09, 17.66)	**0.0096**	0.2219	0.0639
4-weeks follow-up	6.50 (2.01, 10.99)	**0.0004**	12.59 (7.39, 17.80)	0.1515	15.00 (10.53, 19.47)	0.0848	0.0614	**0.0015**
PSQI								
Baseline	14.25 (11.77, 16.73)		12.17 (9.48, 14.86)		13.00 (10.64, 15.36)			
2-weeks post-treatment	11.13 (8.96, 12.29)	**0.0020**	11.55 (8.05, 15.05)	0.6335	12.38 (9.15, 15.60)	0.3884	0.3155	0.1067
4-weeks post-treatment	9.25 (6.33, 12.17)	**0.0066**	10.56 (9.70, 14.42)	0.3632	11.63 (8.87, 14.38)	0.1112	0.2969	0.0556
4-weeks follow-up	8.88 (5.26, 12.49)	**0.0280**	10.12 (5.64, 14.61)	0.2966	11.38 (7.02, 15.72)	0.1719	0.5476	0.1698
Sleep diary (SOL, min)								
Baseline	53.30 (23.60, 83.09)		35.23 (25.41, 45.06)		28.50 (20.41, 36.59)			
2-weeks post-treatment	32.76 (14.19, 51.33)	**0.0216**	30.37 (16.44, 44.31)	0.4192	28.39 (14.91, 41.87)	0.9762	0.3305	0.1500
4-weeks post-treatment	28.64 (9.96, 47.31)	**0.0132**	25.63 (11.84, 39.42)	0.0947	28.21 (15.64, 40.78)	0.9467	0.4041	0.0910
4-weeks follow-up	26.94 (12.65, 41.23)	**0.0186**	24.70 (14.26, 35.15)	**0.0144**	25.09 (10.08, 40.09)	0.1319	0.4421	0.2414
Sleep diary (TST, min)								
Baseline	294.5 (243.2, 345.7)		341.9 (246.2, 437.7)		310.5 (275.7, 345.4)			
2-weeks post-treatment	338.0 (289.1, 386.8)	**0.0348**	343.8 (272.0, 415.7)	0.9152	336.4 (278.2, 394.7)	0.1290	0.2084	0.5124
4-weeks post-treatment	369.1 (306.6, 431.7)	**0.0078**	336.2 (225.8, 446.7)	0.8186	341.1 (289.3, 392.8)	**0.0234**	**0.0072**	0.0919
4-weeks follow-up	378.8 (333.5, 424.0)	**0.0011**	376.0 (314.8, 437.3)	0.0939	338.2 (296.8, 379.7)	0.0920	0.1270	**0.0257**
Sleep diary (SE, %)								
Baseline	70.5 (57.1. 84.0)		80.3 (67.1, 93.4)		74.5 (63.6, 85.4)			
2-weeks post-treatment	80.8 (68.7, 92.9)	**0.0030**	84.4 (73.4, 95.4)	0.1693	78.6 (66.0, 91.1)	0.1807	0.2266	0.1436
4-weeks post-treatment	84.5 (76.2, 92.7)	**0.0073**	84.2 (74.9, 93.5)	0.3884	80.2 (66.9, 93.5)	0.1019	0.2885	0.1396
4-weeks follow-up	87.0 (79.7, 94.3)	**0.0052**	91.0 (86.4, 95.5)	**0.0129**	80.7 (72.2, 89.1)	0.0516	0.8613	**0.0259**

*Abbreviations*: *CI* confidence interval, *ISI* insomnia severity index, *PSQI* Pittsburgh sleep quality index, *SE* sleep efficiency, *SOL* sleep onset latency, *TST* total sleep time

At 4 weeks post-treatment, EA group showed a 5 points reduction of the mean PSQI score compared with baseline (*P* = .0066), with a 1.61 and 1.38 reduction in the sham-EA and usual care groups (*P* = .3632 and *P* = .1112, each). There was no significant between-group difference in PSQI scores during the trial period, although the difference between EA and usual care at 4 weeks post-treatment showed borderline significance (*P* = .0556). All sleep diary-derived variables including SOL, TST, and SE significantly improved only in the EA group at 4 weeks post-treatment. Among them, between-group significance existed in TST between the EA and sham-EA groups at 4 weeks post-treatment (*P* = .0072) and in TST and SE between the EA and usual care groups at 4 weeks’ follow-up (*P* = .0257 and *P* = .0259, each) (Table 2). The actigraphy-derived sleep measures showed no within-group and between-group differences at 4 weeks post-treatment (Table 3). The SE values extracted from actigraphy and the sleep diary showed no significant correlation (Pearson correlation coefficient = .0053, *P* = .9637). No significant within-group and between-group differences were observed in MoCA scores, and salivary levels of cortisol and melatonin. However, the baseline mean MoCA score of each group was 25–26, which means that there was no significant cognitive impairment in people with cancer participating in this study [42]. Therefore, it seems that this resulted in no significant between-group and within-group differences after treatment. The FACT-F scores in EA group showed a significant improvement compared with the sham-EA group at 4 weeks post-treatment (*P* = .0305) (Table 4).

**Table 3:** Actigrahy Sleep Measures

Variable	Electroacupuncture (*N* = 8)		Sham (*N* = 6)		Usual-care (*N* = 8)		*P* value (Electroacupuncture versus sham)	*P* value (Electroacupuncture versus usual-care)
	Mean (95% CI)	*P* value (within group)	Mean (95% CI)	*P* value (within group)	Mean (95% CI)	*P* value (within group)		
SOL, min								
Baseline	8.87 (3.77, 13.98)		8.17 (3.25, 13.08)		6.11 (2.04, 10.17)			
2-weeks post-treatment	21.88 (4.74, 39.01)	0.1135	11.69 (1.66, 21.72)	0.3866	3.60 (0.00, 8.27)	0.3319	0.3186	**0.0355**
4-weeks post-treatment	14.39 (2.58, 26.20)	0.1890	13.09 (0.00, 26.51)	0.4582	3.72 (0.00, 10.15)	0.5018	0.9664	0.1658
4-weeks follow-up	10.26 (4.00, 16.52)	0.5598	5.82 (0.76, 10.87)	0.4052	3.94 (0.79, 7.08)	0.1449	0.3294	0.1908
TST, min								
Baseline	343.43 (275.56, 411.30)		330.93 (263.79, 398.06)		333.48 (293.78, 373.18)			
2-weeks post-treatment	316.00 (265.21, 366.78)	0.4367	323.16 (269.37, 376.96)	0.7577	345.42 (303.40, 387.44)	0.4571	0.7648	0.2775
4-weeks post-treatment	355.39 (297.19, 413.60)	0.5563	325.49 (255.85, 395.13)	0.7738	348.29 (312.16, 384.41)	0.3055	0.4806	0.9820
4-weeks follow-up	361.52 (320.49, 402.55)	0.3543	336.48 (281.51, 391.44)	0.7801	334.33 (297.42, 371.24)	0.9712	0.5070	0.3703
SE, %								
Baseline	80.4 (70.3, 90.6)		78.2 (71.6, 84.9)		78.9 (72.2, 85.7)			
2-weeks post-treatment	74.8 (63.1, 86.5)	0.3685	79.2 (71.0, 87.4)	0.7807	80.6 (72.8, 88.3)	0.5004	0.4539	0.2792
4-weeks post-treatment	81.0 (73.8, 88.2)	0.8603	78.3 (72.0, 84.6)	0.9753	82.0 (75.3, 88.6)	0.0848	0.6762	0.5317
4-weeks follow-up	82.8 (76.1, 89.4)	0.4818	80.5 (73.0, 87.9)	0.4553	79.2 (73.6, 84.9)	0.9255	0.8013	0.4818

*Abbreviations CI* confidence interval, *SE* sleep efficiency, *SOL* sleep onset latency, *TST* total sleep time

**Table 4:** Other Clinical Outcome Measures

Variable	Electroacupuncture (*N* = 8)		Sham (*N* = 6)		Usual-care (*N* = 8)		*P* value (Electroacupuncture versus sham)	*P* value (Electroacupuncture versus usual-care)
	Mean (95% CI)	*P* value (within group)	Mean (95% CI)	*P* value (within group)	Mean (95% CI)	*P* value (within group)		
FACT-F								
Baseline	107.88 (96.87, 118.88)		88.50 (75.25, 101.75)		99.88 (82.21, 117.54)			
4-weeks post-treatment	120.00 (103.35, 136.65)	0.0579	94.58 (81.44, 107.72)	0.2445	106.63 (82.76, 130.49)	0.4331	**0.0305**	0.3111
MoCA								
Baseline	25.38 (23.83, 26.92)		26.17 (23.56, 28.78)		25.13 (22.62, 27.63)			
4-weeks post-treatment	26.63 (23.53, 29.72)	0.3656	25.75 (19.60, 31.90)	0.8240	25.75 (19.60, 31.90)	0.0745	0.7150	0.7658
Saliva melatonin (10 PM)								
Baseline	5.43 (0.00, 11.90)		2.59 (1.15, 4.04)		4.07 (1.54, 6.59)			
4-weeks post-treatment	4.42 (0.69, 8.15)	0.4477	3.02 (0.00, 6.69)	0.5661	6.09 (0.00, 12.72)	0.4046	0.8401	0.3240
Saliva melatonin (7 AM)								
Baseline	9.15 (2.14, 16.15)		3.81 (1.93, 5.70)		3.96 (0.00, 7.92)			
4-weeks post-treatment	5.71 (1.49, 9.93)	0.2848	4.18 (2.85, 5.51)	0.8083	6.83 (0.00, 14.70)	0.1508	0.8161	0.3120
Saliva cortisol (10 PM)								
Baseline	0.058 (0.024, 0.091)		0.057 (0.004, 0.111)		0.042 (0.018, 0.067)			
4-weeks post-treatment	0.081 (0.009, 0.153)	0.5387	0.043 (0.018, 0.068)	0.4814	0.071 (0.002, 0.140)	0.3899	0.7292	0.3510
Saliva cortisol (7 AM)								
Baseline	0.293 (0.105, 0.482)		0.219 (0.077, 0.361)		0.246 (0.098, 0.394)			
4-weeks post-treatment	0.239 (0.098, 0.380)	0.3571	0.243 (0.066, 0.421)	0.4703	0.214 (0.082, 0.346)	0.5667	0.9884	0.4816

*Abbreviations*: *CI* confidence interval, *FACT-F* functional assessment of cancer therapy-fatigue, *MoCA* Montreal cognitive assessment

### Safety

Twenty-three AEs were reported over 185 visits, with no significant between-group difference in the incidence of AEs (10 cases/96 visits [10.42%] in the EA group, including 2 cases of headache and 1 case each of cough, lumbago, common cold, enteritis, dizziness, knee joint pain, rhinitis, and dyspepsia; 7 cases/57 visits [12.28%] in the sham-EA group, including 2 cases of common cold and 1 case each of shoulder joint pain, skin allergy, lymphadenitis, hematuria, and dyspepsia; and 6 cases/32 visits [18.75%] in the usual care group, including 1 case each of arthritis, skin spot, diarrhea, dyspepsia, toothache, and ileus; Fisher’s exact test, *P* = .4323). One participant in the usual care group was hospitalized for 10 days due to postoperative ileus during the trial period, but it was judged not to be relevant to our study. All AEs were considered to be unrelated to our study and subsided spontaneously.

### Blinding and credibility test

At week 1, 7 of 8 participants in the EA group (new BI 0.875; 95% CI 0.646 to 1.104) versus 5 of 6 participants in the sham-EA group (new BI -0.667; 95% CI − 1.263 to − 0.070) guessed their treatment to be real EA. At 4 weeks post-treatment, the numbers were 8/8 participants in the EA group (new BI 1; 95% CI 1 to 1) versus 3/4 participants in the sham-EA group (new BI -0.750; 95% CI − 1.174 to − 0.326). Thus, blinding was well-maintained in the sham-EA group during the trial period. There were no significant differences in the level of confidence in the treatment the participants received between before and after the study in the both EA and sham-EA groups. In addition, the mean difference before and after treatment generally showed no significant difference between the 2 groups (Additional file 1).

## Discussion

This study showed that EA significantly improved sleep quality measured by ISI, PSQI, and sleep diary-derived SOL, TST, and SE in people with cancer-related insomnia without any serious AEs, although there were no significant between-group differences at 4 weeks post-treatment, our primary endpoint. However, there were significant differences in ISI scores between the EA and usual care group at 4 weeks’ follow-up. Since the minimal clinically important differences (MCIDs) of ISI and PSQI scores in people with insomnia are 6 and 3 points, respectively [43, 44], the mean reductions of 10.13 and 5.00 in ISI and PSQI scores at 4 weeks post-treatment showed that EA had a clinically significant benefit in improving sleep quality in people with insomnia.

There was borderline significant difference between EA and usual care groups at 4 weeks post-treatment in the ISI (*P* = .0639) and PSQI scores (*P* = .0556) and a significant difference between them at 2 weeks post-treatment (*P* = .0481) and 4 weeks’ follow-up (*P* = .0015) in the ISI score. However, the ISI score at 4 weeks post-treatment was not significantly different between the EA and sham-EA groups (*P* = .2219). We used a validated non-penetrating sham control at heterosegmental non-acupoints to minimize the relevant bias and maintained blinding and credibility in the sham-EA group during the trial period. However, unlike placebo pills, the tactile component of sham acupuncture may produce biophysical effects such as evoking the somatosensory system, strengthening the doctor-patient relationship, and enhancing the patient’s general condition, which are important factors influencing the treatment of insomnia in actual clinical practice [45]. Thus, these are not necessarily inert interventions [46, 47] and therefore, similar to other studies using sham control groups [23, 48], this unavoidable characteristic of the sham acupuncture would have made an effect difference between EA and sham-EA smaller than that between EA and usual care in this study. Interestingly, only the EA group continued to improve significantly at 4 weeks’ follow-up in ISI score, showing significant and borderline differences compared to the usual care (*P* = .0015) and sham-EA (*P* = .0614). These results suggest the possibility of lasting effects of EA.

Mao et al. showed that 8 weeks of EA did not improve insomnia and significantly improved only fatigue [49], contradicting our study. However, they targeted breast cancer participants with aromatase inhibitor-related arthralgia and assessed sleep patterns additionally. Because our study targeted people with cancer-related insomnia, the substantial differences in participant baseline data preclude direct comparisons of the two studies. Feng et al. revealed that acupuncture can significantly reduce malignancy-related depression and improve the sleep quality of people with cancer [50]. However, unlike our study comparing EA with sham-EA or usual care, they targeted people with cancer with both depression and insomnia and compared acupuncture with Fluoxetine.

The effectiveness of acupuncture for insomnia has been evidenced in multiple studies [23, 51]. However, to the best of our knowledge, this is the first exploratory study comparing EA with sham-EA and usual care for insomnia in people with cancer. Insomnia is more common and longer-lasting in people with cancer than in the general population and it affects the natural history of tumors [3, 5, 7]. Furthermore, the effectiveness of hypnotics lacks evidence [12]. Our results showed improved sleep quality and high adherence and completion rates, suggesting that EA can be used for treating cancer-related insomnia without any serious AEs. In addition, the blinding and credibility tests showed successful results in the sham-EA group. This is particularly encouraging, given the fact that people in Korea are very familiar with acupuncture.

Many studies have attempted to elucidate the mechanisms underlying acupuncture, including neural regulation such as modulating the imbalance between parasympathetic and sympathetic activity, endocrine regulation, and immunological regulation such as improving T-lymphocyte and natural killer cell function [52, 53]. Some studies have suggested that acupuncture in people with cancer may alleviate the adverse reactions caused by chemotherapy or radiotherapy [54]. Although there have been previous studies showing that acupuncture can improve insomnia by affecting cortisol and melatonin secretion [37–39], in our study there was no change in salivary melatonin and cortisol levels before and after treatment. However, since we included only a small number of patients, larger trials will be needed to clarify these conflicting results.

This study has several limitations. First, we originally planned to enrol a total of 45 participants. However, only 22 participants were included, despite multicenter recruitment for more than a year via online and offline advertisements. However, since this is a pilot study to obtain information such as recruitment rates, determine the appropriateness of the inclusion and exclusion criteria, and assess the feasibility, these results may yield useful information for future confirmatory clinical trials. Only 28 participants were screened for eligibility during the trial period, but the adherence and completion rates of the enrolled participants were high. This can be attributed to the difficulties in participant recruitment as a result of the strict inclusion and exclusion criteria, given the various characteristics of people with cancer. Our study was conducted for people with moderate to severe insomnia with an ISI score of 15 or higher, but in a recent study comparing acupuncture with CBT-I for cancer-related insomnia [55], participants with ISI scores greater than 7 were included. Additionally, inclusion of only those who completed cancer-related treatment at least 12 weeks prior to the screening visit may have been an excessively strict criterion since most people with cancer are receiving ongoing cancer-related treatment. Therefore, we suggest shortening this period to 1 month in future studies, on the basis of a recent related study [55]. According to a recent clinical trial targeting the general insomnia population, in which EA was performed 10 times for 4 weeks similar to this study, a total of 150 participants were enrolled over 9 months in 4 Korean medicine hospitals [56]. Taking this into account, the participant recruitment in our study seems to have been very slow because our study was conducted with people with cancer. In addition, our study recruited participants at a general university affiliated with Korean medicine hospitals in Korea, which are not specialized to treat cancer patients. In Korea, there are differences in the characteristics of each region and clinical institution, so our study provides information on this as a feasibility trial. Therefore, in future clinical trials, a significant change in recruitment strategy is required, such as recruiting participants from cancer centers that specialize in treating cancer patients. Second, we could not conduct subgroup analyses according to the severity of insomnia, blood deficiency scoring system, or treatment expectation to the EA at baseline as described in our protocol [21] because of the small number of participants included. Finally, there was low correlation between the sleep diary and actigraphy-derived SE values, consistent with previous studies showing better estimates of sleep with actigraphy [23, 57]. This might be because actigraphy records lying awake without moving as sleeping. The discrepancy in these values suggests that additional objective measures such as polysomnography may be helpful for evaluating the sleep quality more accurately in future studies.

## Conclusions

The current study showed high adherence to EA for people with cancer-related insomnia by using a validated placebo control and validated subjective and objective sleep measures. Additional confirmatory trials with formal sample size calculations, longer follow-up, and a substantial change to the recruitment procedure should be performed to confirm the current findings.

## Supplementary Information

**Additional file 1.** The results of blinding and credibility test.

## Data Availability

The data generated during and/or analyzed during the current study are available from the corresponding author on reasonable request.

## Abbreviations

AE: Adverse event
ANCOVA: Analysis of covariance
BI: Blinding index
CBT-I: Cognitive Behavior Therapy for Insomnia
CI: Confidence interval
EA: Electroacupuncture
FACT-F: Functional Assessment of Cancer Therapy-Fatigue
free T4: Free thyroxine
ISI: Insomnia Severity Index
MCID: Minimal clinically important difference
MoCA: Montreal Cognitive Assessment
NCI: National Cancer Institute
PSQI: Pittsburgh Sleep Quality Index
RCT: Randomized controlled trial
SE: Sleep efficiency
SOL: Sleep-onset latency
TIB: Total time in bed
TSH: Thyroid-stimulating hormone
TST: Total sleep time
