# Supplementary material for: Electroacupuncture for treating cancer-related insomnia: a multicenter, assessor-blinded, randomized controlled, pilot clinical trial
Source: BMC Complement Med Ther. 2022 Mar 18;22:77. doi: 10.1186/s12906-022-03561-w (PMC8932204; doi:10.1186/s12906-022-03561-w)
Supplement: Supplementary file 1 — Additional file 1. The results of blinding and credibility test. [file 12906_2022_3561_MOESM1_ESM.docx]

**Additional file 1**. The results of blinding and credibility test

1. Blinding test

| Week 1 | Electroacupuncture | Sham |
| --- | --- | --- |
| Real electroacupuncture | 7 | 5 |
| Sham-electroacupuncture | 0 | 1 |
| Don’t know | 1 | 0 |
| New Blind Index | 0.875 (0.646, 1.104) | -0.667 (-1.263, -0.070) |

| 4-week post-treatment | Electroacupuncture | Sham |
| --- | --- | --- |
| Real electroacupuncture | 8 | 3 |
| Sham-electroacupuncture | 0 | 0 |
| Don’t know | 0 | 1 |
| New Blind Index | 1.000 (1.000, 1.000) | -0.750 (-1.174, -0.326) |

1. Credibility test

| Credibility Assessment | Electroacupuncture | Sham | Mean difference | *P* value |
| --- | --- | --- | --- | --- |
| A |  |  |  |  |
| Week 1 | 5.00 (3.91, 6.09) | 5.17 (4.74, 5.60) |  |  |
| 4-week post-treatment | 4.76 (3.51, 5.99) | 3.75 (0.74, 6.76) | 1.26 (-0.38, 2.90) | 0.1166 |
| Difference | -0.25 (-0.84, 0.34) | -1.50 (-4.26, 1.26) |  |  |
| *P* value | 0.3506 | 0.1817 |  |  |
| B |  |  |  |  |
| Week 1 | 4.88 (3.83, 5.92) | 4.67 (4.12, 5.21) |  |  |
| 4-week post-treatment | 4.88 (3.66, 6.09) | 4.25 (1.86, 6.64) | 0.75 (-0.77, 2.27) | 0.2923 |
| Difference | 0.00 (-0.63, 0.63) | -0.75 (-3.14, 1.64) |  |  |
| *P* value | 0.9999 | 0.3910 |  |  |
| C |  |  |  |  |
| Week 1 | 4.88 (3.74, 6.01) | 4.17 (3.38, 4.96) |  |  |
| 4-week post-treatment | 5.13 (4.18, 6.07) | 3.50 (0.74, 6.26) | 1.04 (0.06, 2.02) | 0.0392 |
| Difference | 0.25 (-0.14, 0.64) | -0.75 (-2.27, 0.77) |  |  |
| *P* value | 0.1705 | 0.2152 |  |  |
| D |  |  |  |  |
| Week 1 | 5.29 (4.59, 5.98) | 4.50 (3.93, 5.07) |  |  |
| 4-week post-treatment | 4.63 (3.63, 5.62) | 4.00 (1.75, 6.25) | 0.27 (-1.65, 2.19) | 0.7504 |
| Difference | -0.43 (-1.33, 0.47) | -0.50 (-2.55, 1.55) |  |  |
| *P* value | 0.2894 | 0.4950 |  |  |

A: “How effective do you think this treatment might be for insomnia?”

B: “How acceptable is the treatment?”

C: “How confident would you be recommending this treatment to a friend who experiences insomnia?”

D: “How successful do you think this treatment would be in reduction of other symptoms?”
